# Effect of insulin degludec versus sitagliptin in patients with type 2 diabetes uncontrolled on oral antidiabetic agents

**DOI:** 10.1111/dom.12115

**Published:** 2013-05-06

**Authors:** A Philis-Tsimikas, S Del Prato, I Satman, A Bhargava, M Dharmalingam, T V Skjøth, S Rasmussen, A J Garber

**Affiliations:** 1 Scripps Whittier Diabetes InstituteSan Diego, CA, USA; 2 Department of Endocrinology and Metabolism, Section of Metabolic Diseases, University of PisaPisa, Italy; 3 Division of Endocrinology and Metabolism, Department of Internal Medicine, Istanbul Medical Faculty, Istanbul UniversityIstanbul, Turkey; 4 Iowa Diabetes & Endocrinology Research CenterDes Moines, IA, USA; 5 Bangalore Endocrinology & Diabetes Research CenterMalleswaram, Bangalore, India; 6 Medical & Science, Novo Nordisk A/SSøborg, Denmark; 7 Biostatistics, Novo Nordisk A/SSøborg, Denmark; 8 Division of Endocrinology, Diabetes and Metabolism, Baylor College of MedicineHouston, TX, USA

**Keywords:** efficacy, insulin degludec, safety, sitagliptin, type 2 diabetes

## Abstract

**Aim:**

The efficacy and safety of insulin degludec (IDeg), a new basal insulin with an ultra-long duration of action, was compared to sitagliptin (Sita) in a 26-week, open-label trial.

**Methods:**

Insulin-naïve subjects with type 2 diabetes [n = 458, age: 56 years, diabetes duration: 7.7 years, glycosylated haemoglobin (HbA1c):8.9% (74 mmol/mol)] were randomized (1:1) to once-daily IDeg or Sita (100 mg orally) as add-on to stable treatment with 1 or 2 oral antidiabetic drugs (OADs).

**Results:**

Superiority of IDeg to Sita in improving HbA1c and fasting plasma glucose (FPG) was confirmed [estimated treatment difference (ETD) IDeg–Sita for HbA1c: −0.43%-points [95% confidence interval (CI): −0.61; −0.24, p < 0.0001] and for FPG: −2.17 mmol/l (95% CI: −2.59; −1.74, p < 0.0001)]. HbA1c < 7% (<53 mmol/mol) was achieved by 41% (IDeg) versus 28% (Sita) of patients, estimated odds ratio IDeg/Sita: 1.60 (95% CI: 1.04; 2.47, p = 0.034). There was no statistically significant difference in the rate of nocturnal confirmed hypoglycaemia between IDeg and Sita [0.52 vs. 0.30 episodes/patient-year, estimated rate ratio (ERR): IDeg/Sita: 1.93 (95% CI: 0.90; 4.10, p = 0.09)]. Rates of overall confirmed hypoglycaemia were higher with IDeg than with Sita [3.1 vs. 1.3 episodes/patient-year, ERR IDeg/Sita: 3.81 (95% CI: 2.40; 6.05, p < 0.0001)]. IDeg was associated with a greater change in body weight than Sita [ETD IDeg–Sita: 2.75 kg (95% CI: 1.97; 3.54, p < 0.0001)]. The overall rates of adverse events were low and similar for both groups.

**Conclusions:**

In patients unable to achieve good glycaemic control on OAD(s), treatment intensification with IDeg offers an effective, well-tolerated alternative to the addition of a second or third OAD.

## Introduction

A range of long-term studies have highlighted the importance of good glycaemic control to avoid or delay late-stage diabetes complications 1–4. Early treatment strategies beyond lifestyle changes and metformin include adding one or more oral antidiabetic drugs (OADs), incretin-based therapies or basal insulin. Once lifestyle changes and metformin treatment have been initiated, various factors influence the order in which further pharmaceutical interventions are prescribed during the course of diabetes treatment. Efforts have been made over the years 5–8 to create algorithms for diabetes management; however, it is becoming clearer that individualized treatment regimens are necessary for effective diabetes management 9. Navigating through the multitude of treatment choices can, however, be difficult for physicians, especially considering the lack of comparative studies available targeting specific patient populations at various stages of disease progression.

In general, initiation of insulin therapy is often postponed and only added after two or more OADs and/or glucagon-like peptide-1 (GLP-1) receptor agonists have proven inadequate to achieve or maintain glycaemic control 7,9,10. However, safer and more user-friendly insulin preparations could be used at an earlier stage in a more advantageous manner to aid patients in achieving glucose control.

Insulin degludec (IDeg) is a new basal insulin that uses a novel mechanism of protraction resulting in a flat, stable profile and with a duration of action greater than 42 h 11. IDeg provides substantial reductions in glycosylated haemoglobin (HbA1c) with low rates of overall and nocturnal hypoglycaemia, and is the only basal insulin with the potential of being dosed at any time of day with the possibility of changing injection time in response to changes in patients’ daily schedules, without compromising efficacy or safety 12–15.

In this comparative study between the basal insulin IDeg and the dipeptidyl peptidase-4 (DDP-4) inhibitor sitagliptin (Sita), we investigated the differences in efficacy and safety between these two treatment options. The patient population comprised insulin-naïve subjects with type 2 diabetes currently treated with 1–2 OADs qualifying for intensified treatment.

## Materials and Methods

### Design

This was a confirmatory, 26-week, randomized, open-label, multicentre, multinational, controlled trial comparing the efficacy and safety of IDeg and Sita in insulin-naïve adult subjects with type 2 diabetes for ≥6 months. Eligible subjects had a body mass index (BMI) limit of ≤40 kg/m^2^, HbA1c of 7.5–11.0% (58–97 mmol/mol) (both inclusive) [7.5–10% (58–86 mmol/mol) both inclusive for Argentina] and were treated with 1–2 OADs, including metformin, sulphonylureas (SUs), glinides or pioglitazone (Pio), in any combination with an unchanged dose for at least 3 months. Patients were excluded if they were using a GLP-1 receptor agonist, another DPP-4 inhibitor or rosiglitazone within 3 months of screening.

Subjects were recruited from 78 sites in seven countries (Argentina, Canada, India, Mexico, South Africa, Turkey and USA) and provided written informed consent.

The study was approved by local ethics committees and health authorities and carried out in accordance with International Conference on Harmonisation (ICH) Good Clinical Practice guidelines 16 and the Declaration of Helsinki 17. The trial is registered at clinicaltrials.gov (ID number: NCT01046110).

### Treatment

Through a central interactive voice/web-response system, eligible participants were randomly allocated 1:1 to receive IDeg once daily (100 units/ml, 3 ml Flexpen® Novo Nordisk, Bagsværd, Denmark) or Sita (100 mg, tablet; Januvia®, Merck & Co. Inc., Whitehouse Station, NJ, USA) as add-on to treatment with 1 or 2 OADs (metformin, SU, glinides or Pio). Participants were stratified according to the use of Pio at screening, and the stratification was employed to ensure an approximately equal distribution of Pio users in the two treatment arms. The trial was open-label because of the distinctive difference in the mode of trial product administration. IDeg was injected once daily at any time of day, between waking up and bedtime, as long as there was a minimum of 8 and a maximum of 40 h maintained between injections. Sita 100 mg was administered orally once daily. IDeg was titrated weekly applying a treat-to-target approach to achieve a pre-breakfast self-measured plasma glucose (SMPG) target of <5.0 mmol/l (90.0 mg/dl). Plasma glucose (PG) was measured by the subjects at home using a blood glucose meter that included plasma calibrated test strips converting blood glucose values to PG values. The insulin starting dose was 10 units, and based on the average of three consecutive pre-breakfast SMPG values before each visit, a new dose was recommended in accordance with a titration algorithm (Table 1) via an electronic data capture system. The investigator could accept or reject the recommended dose. The subjects recorded in their diary every day throughout the trial if the IDeg injection was taken in the morning (between getting up and breakfast), daytime (between breakfast and main evening meal) or in the evening (between main evening meal and bedtime). The Safety Committee from the sponsor (Novo Nordisk) that performed ongoing safety surveillance and the external cardiovascular Event Adjudication Committee (EAC) were masked to treatment. All Novo Nordisk staff involved in data handling were masked to participants’ treatment allocation until dataset was locked for statistical analysis.

**Table 1:** Titration algorithm for basal insulin dose

Pre-breakfast plasma glucose*		
mmol/l	mg/dl	Adjustment of insulin degludec (units)
<5.0	<90	No adjustment
<7.0	<126	+2
<8.0	<144	+4
<9.0	<162	+6
≥9.0	≥162	+8

Doses were decreased by 2 units (or 5% reduction if dose >45 units) if FPG was 3.1–3.9 mmol/l (56–71 mg/dl), and by 4 units (or 10% reduction if dose >45 units) if FPG was <3.1 mmol/l (<56 mg/dl). FPG, fasting plasma glucose.

* Mean of three consecutive days’ measurements.

### Trial endpoints

The primary endpoint was change from baseline in HbA1c after 26 weeks of treatment. Secondary efficacy endpoints included change from baseline in central-laboratory-measured fasting plasma glucose (FPG) after 26 weeks of treatment, frequency of responders for HbA1c (<7.0%; <53 mmol/mol) at end of trial, responders for HbA1c (<7.0%) at end of trial without confirmed hypoglycaemic episodes, mean SMPG (mean of 9-point profile is defined as the area under the profile using the trapezoidal method divided by the measurement time), prandial PG increment from SMPG 9-point profile (prandial increment was defined as the difference in SMPG 90 min before and after a meal), frequency of responders for HbA1c (<6.5%; 48 mmol/mol) at the end of the trial and health-related quality of life (HRQoL).

Safety assessments included adverse events (AEs), hypoglycaemic episodes, insulin dose, physical examination, body weight, vital signs, fundoscopy, electrocardiogram (ECG) and laboratory tests. Confirmed hypoglycaemia was defined as hypoglycaemic episodes with a PG value of <3.1 mmol/l (56 mg/dl) (regardless of symptoms) or severe episodes where assistance from another person was required. Confirmed hypoglycaemic episodes occurring between 00:01 hours and 05:59 hours (inclusive) were classified as nocturnal, and those occurring between 06:00 hours and 00:00 hours (inclusive) as diurnal. Cardiovascular events were reviewed by an independent EAC. Laboratory analyses were performed at Quintiles Central Laboratories in Argentina, India, Scotland, Singapore, South Africa, Switzerland and USA. The following validated questionnaires assessed HRQoL outcomes: Diabetes Medication Satisfaction Questionnaire (DiaMedSat), Diabetes Productivity (DPM), Short-Form 36 Health Survey version 2 (SF-36 v2), Treatment-Related Impact Measure—Diabetes (TRIM–D) and Hypoglycaemic Episode—Interview Questionnaire.

### Statistical methods

All statistical analyses were performed using sas version 9.1.3 (SAS Institute, Cary, NC, USA). This trial’s primary objective was to confirm the superiority of IDeg to Sita as assessed by change in HbA1c from baseline to after 26 weeks. Superiority was considered confirmed if the upper bound of the two-sided 95% confidence interval (CI) was <0%. Type I error was controlled by adopting a hierarchical (fixed-sequence) testing procedure for selected endpoints in the following order: change in HbA1c, change in FPG, HbA1c responders [<7% (<53 mmol/mol)] and responders (<7%) without hypoglycaemia. The sample size was determined based upon this primary objective using a t-statistic under the assumption of a one-sided test of size 2.5% and a 0.4% mean treatment difference and standard deviation (s.d.) estimate of 1.3% for HbA1c.

Subjects in the full analysis set (FAS), defined as all subjects randomly allocated to treatment were included in the statistical assessments of all efficacy endpoints and treatment comparisons of hypoglycaemia, body weight and lipids. Other safety endpoints were evaluated in subjects exposed to treatment (safety analysis set). A single site was closed down and subjects from this site were excluded from the FAS before unblinding of treatment groups. These subjects were included in the safety analysis set. Missing values were imputed using the last observation carried forward (LOCF) method. Treatment difference in changes in HbA1c from baseline after 26 weeks were assessed using an analysis of covariance (ancova) model, with treatment, antidiabetic therapy at screening, sex and region as fixed factors, and age and baseline value as covariates.

Treatment difference as change from baseline in FPG, body weight, prandial increments and mean PG (based on the 9-point SMPG profile) and HRQoL were analysed using an ancova method similar to that used for the primary endpoint with appropriate baseline adjustments. The number of treatment-emergent confirmed hypoglycaemic episodes per subject-year of exposure was analysed using a negative binomial regression model including treatment, antidiabetic therapy at screening, sex and region as fixed factors and age as covariate, using all reported treatment-emergent episodes. HbA1c responders were analysed using a logistic regression adjusted for the same variables as in the primary model.

## Results

### Demographic and baseline characteristics of the subjects

**Figure 1:**
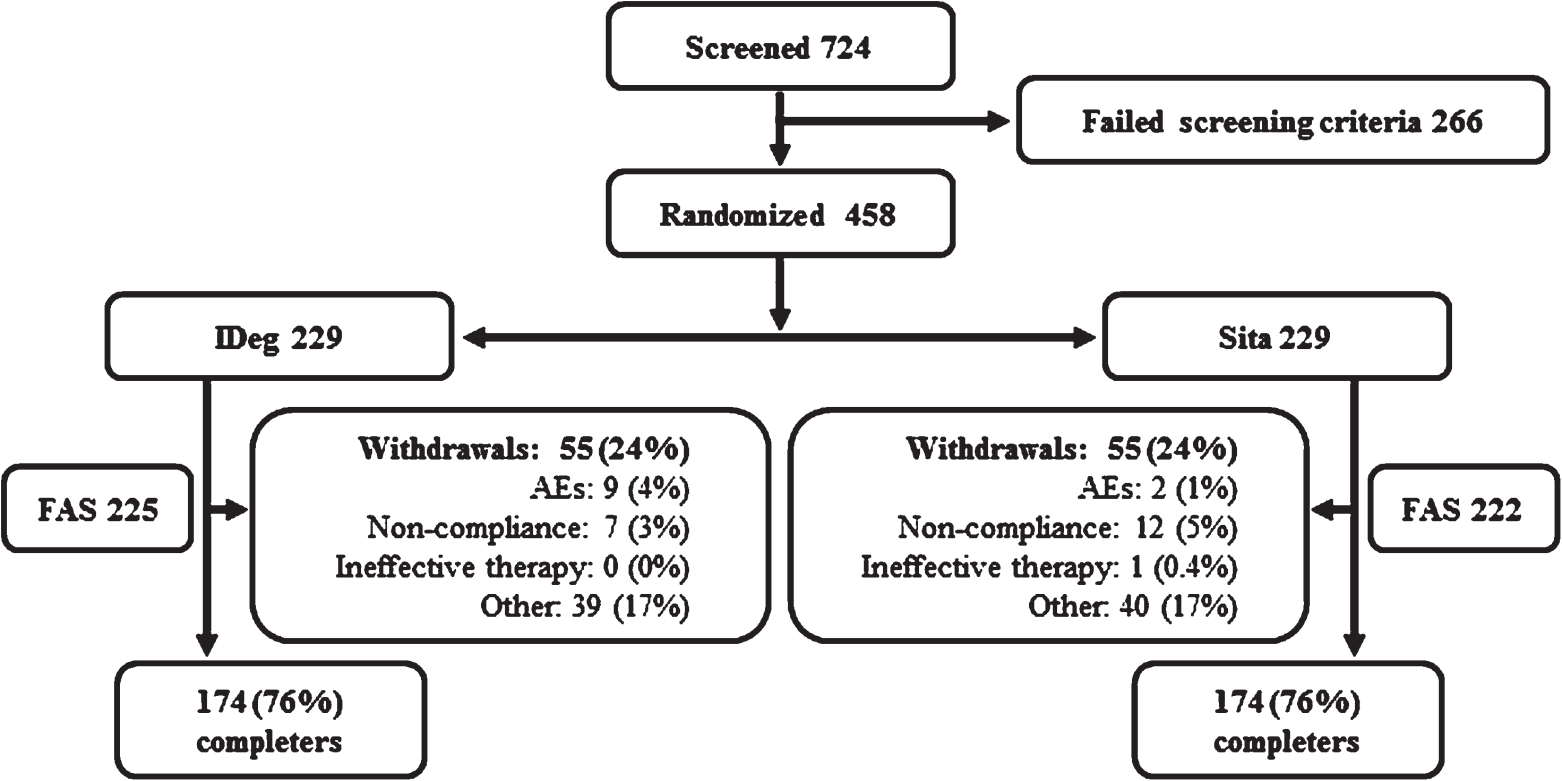
Patient flow. ‘Other’ included subjects who withdrew consent, subjects who relocated, subjects who were lost to follow-up and subjects who discontinued due to meeting withdrawal criteria. AE, adverse event; FAS, full analysis set; IDeg, insulin degludec; OD, once daily; SAS, safety analysis set; Sita, sitagliptin.

There were no clinically relevant differences in baseline or demographic characteristics between subjects in the two treatment groups (Table 2). A total of 724 subjects were screened, of whom 458 were randomized. Consistent with the 1:1 randomization scheme, 229 subjects were then assigned to treatment with either IDeg or Sita, and 174 (76%) of these subjects completed the trial in each treatment group. Withdrawal patterns were similar between groups (figure 1).

**Table 2:** Baseline and demographic characteristics of the two treatment groups

	IDeg	Sita
Full analysis set, n	225	222
Female/male, %	37.3/62.7	45.5/54.5
Race: White/Black/Asian/ other, %	60.0/7.6/25.3/7.1	62.6/7.7/24.8/5.0
Ethnicity: Hispanic or Latin American, %	20.0	22.1
Age, years	56.4 (±10.2)	54.9 (±11.4)
Weight, kg	83.9 (±19.3)	86.1 (±19.8)
BMI (kg/m^2^)	30.0 (±5.1)	30.8 (±5.2)
Duration of diabetes, years	7.8 (±6.2)	7.7 (±5.9)
HbA1c, %	8.8 (±1.0)	9.0 (±1.0)
HbA1c, mmol/mol	72.7	74.9
FPG, mmol/l (mg/dl)	9.4 (±2.6) [169.4 (±46.8)]	9.9 (±3.1) [178.4 (±55.9)]
Antidiabetic treatment at screening, n (%)*		
Metformin monotherapy	55 (24.4)	57 (25.7)
Pio ± (SU or glinide) or metformin	9 (4.0)	15 (6.8)
SU or glinide ± metformin	161 (71.6)	150 (67.6)

BMI, body mass index; FPG, fasting plasma glucose; HbA1c, glycosylated haemoglobin; IDeg, insulin degludec; Pio, pioglitazone; Sita, sitagliptin; SU, sulphonylurea.

* Safety analysis set.

### Efficacy

**Figure 2:**
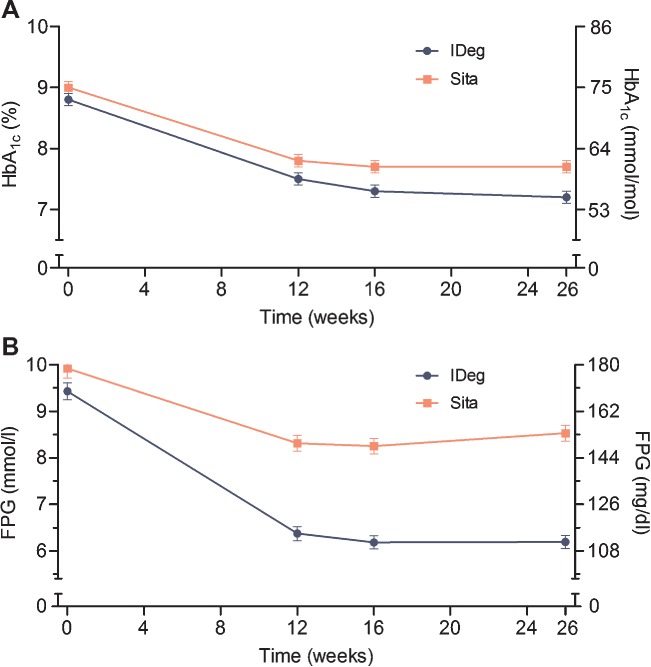
Mean glycosylated haemoglobin (HbA1c) (A) and fasting plasma glucose (FPG) (B) during 26 weeks of treatment. IDeg, insulin degludec; Sita, sitagliptin.

IDeg effectively improved glycaemic control and was superior to Sita in terms of lowering HbA1c after 26 weeks of treatment (figure 2A). After 26 weeks, the observed mean HbA1c was 7.2% (55 mmol/mol) with IDeg and 7.7% (61 mmol/mol) with Sita. The estimated mean change from baseline was −1.52% with IDeg and −1.09% with Sita; the estimated treatment difference (ETD; IDeg–Sita) of −0.43% (−0.61;−0.24)_95% CI_ confirmed the superiority of IDeg to Sita. The supportive analyses including change in FPG and HbA1c responders (<7%) also showed superiority of IDeg. After 26 weeks, the observed mean FPG was 6.2 mmol/l (111.7 mg/dl) with IDeg and 8.5 mmol/l (153.2 mg/dl) with Sita. The estimated mean change from baseline was −3.41 mmol/l (−61.4 mg/dl) with IDeg and −1.24 mmol/l (−22.3 mg/dl) with Sita; the ETD (IDeg–Sita) was −2.17 mmol/l (−2.59; –1.74)_95% CI_ [−39.1 mg/dl (−46.7; −31.4)_95% CI_] (figure 2B). Treatment with IDeg showed a higher proportion of subjects achieving HbA1c < 7.0% (<53 mmol/mol) at end of trial; 41% (IDeg) versus 28% (Sita) of subjects, estimated odds ratio (OR) (IDeg/Sita) 1.60 (1.04; 2.47)_95% CI_.

**Figure 3:**
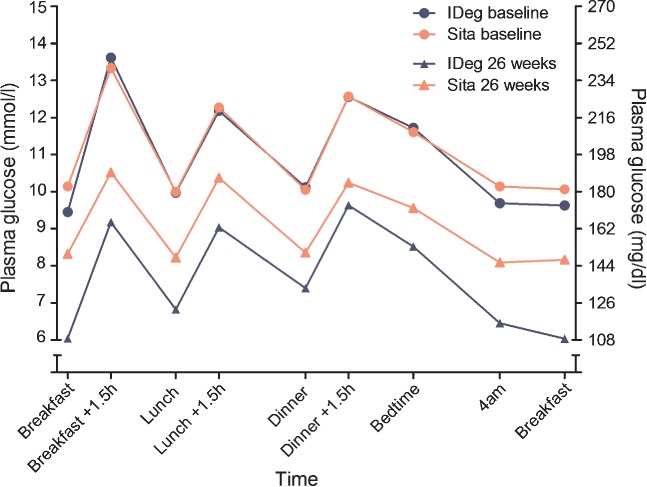
Nine-point self-measured plasma glucose profile. IDeg, insulin degludec; Sita, sitagliptin.

The proportion of subjects achieving HbA1c < 7.0% (<53 mmol/mol) without hypoglycaemia at end of trial was 25% (IDeg) versus 23% (Sita), estimated OR (IDeg/Sita) 0.92 (0.55; 1.53)_95% CI_. The observed proportion of subjects achieving HbA1c ≤ 6.5% (≤48 mmol/mol) at end of trial was 28.0% with IDeg and 14.9% with Sita. The odds of achieving this target were 98% higher with IDeg compared with Sita [estimated OR (IDeg/Sita) 1.98 (1.17; 3.33)_95% CI_]. At all time-points in the 9-point profile, the estimated mean SMPG value was lower for IDeg compared to Sita after 26 weeks of treatment (figure 3). The estimated mean of the overall 9-point profile was lower with IDeg than with Sita; the ETD (IDeg–Sita) was −1.31 mmol/l (−1.69; −0.94)_95% CI_ [−23.60 mg/dl (−30.45; −16.94)_95% CI_] (figure 3). The prandial glucose increment, defined as the difference in SMPG values 90 min before and after a meal, was seen to be higher with IDeg compared to Sita across ‘all meals’ and at breakfast after 26 weeks; the ETD (IDeg–Sita) was 0.35 mmol/l, (0.05; 0.65)_95% CI_ [6.31 mg/dl (0.90; 11.71)_95% CI_] for ‘all meals’ and 0.54 mmol/l, (0.07; 1.02)_95% CI_ [9.73 mg/dl (1.26; 18.38)_95% CI_] for breakfast. The change in nocturnal PG was greater with IDeg than with Sita from bedtime to breakfast; the ETD (IDeg−Sita) was −0.94 mmol/l (−1.43; −0.46)_95% CI_ [−16.94 mg/dl (−25.77; −8.29)_95% CI_]. Mean insulin dose for IDeg increased throughout the trial, most rapidly during the first 16 weeks. The mean daily IDeg dose after 26 weeks was 43 units (0.50 unit/kg). The mean and median difference between the dose according to the titration algorithm and the prescribed dose was close to 0 units throughout the trial for the IDeg group, indicating a close adherence to the titration algorithm. At baseline, approximately half of the subjects took their injection in the morning, approximately one third of the subjects in the evening and the remaining subjects during the day. Approximately 42.0% of the subjects changed their time of injection at least once during the trial, 20% of the subjects changed 1–2 times and 22% changed more than three times. Subjects were advised to take Sita once daily. However, timing of when Sita was taken by the subjects was not recorded. The patient-reported outcome (PRO) results appeared to be similar between the two treatment groups for the DPM, SF-36 v2 and Hypoglycaemic Episode—Interview Questionnaire, with only marginal changes over time. The perceived treatment burden improved less with IDeg compared with Sita based on the TRIM-D and DiabMedSat questionnaire. The results from the TRIM-D questionnaire analysis showed that the ETD after 26 weeks was −4.2 (−7.7; −0.7)_95% CI_. On the basis of the DiabMedSat questionnaire, the improvement in overall treatment satisfaction was smaller with IDeg compared to Sita [ETD −2.7 (−4.8; −0.5)_95% CI_].

### Safety and tolerability

#### Hypoglycaemia

As expected, the rate of confirmed hypoglycaemic episodes was higher with IDeg compared with Sita but there was no difference between treatment groups in the rate of nocturnal confirmed hypoglycaemic episodes. No statistical analysis was performed for the number of severe hypoglycaemic episodes as only one episode was reported in the trial (Table 3). No episodes of nocturnal severe hypoglycaemia were reported during the trial. The rate of confirmed hypoglycaemic episodes in both treatment groups was clearly influenced by concomitant OADs, as lower rates for confirmed hypoglycaemia were seen in the IDeg group and there were no hypoglycaemic episodes in the Sita group in those subjects who were not treated with an SU/glinide (Table 4).

**Table 3:** Hypoglycaemia in the two treatment groups

	IDeg (n = 226)			Sita (n = 228)		
	n	%	R	n	%	R
Severe	1	0.4	0.01	0	0.0	0.00
Confirmed	96	42.5	3.07	29	12.7	1.26
Nocturnal confirmed	29	12.8	0.52	13	5.7	0.30

Safety analysis set; IDeg, insulin degludec; n, number of patients with events; R, rate of hypoglycaemia in episodes per patient-years of exposure; Sita, sitagliptin; %, proportion of patients with events.

**Table 4:** Hypoglycaemia in the comparator group, split according to OAD regimen composition

	Treatment including SU/Pio						Treatment without SU/Pio					
	IDeg (n = 170)			Sita (n = 170)			IDeg (n = 56)			Sita (n = 58)		
	n	%	R	n	%	R	n	%	R	n	%	R
Severe	1	0.6	0.01	0	0.0	0.0	0	0.0	0.0	0	0.0	0.00
Confirmed	86	50.6	3.43	29	17.1	1.71	10	17.9	1.92	0	0.0	0.00
Nocturnal confirmed	25	14.7	0.57	13	7.6	0.40	4	7.1	0.38	0	0.0	0.00

Safety analysis set; IDeg, insulin degludec; n, number of patients with events; OAD, oral antidiabetic drug; Pio, pioglitazone; R, rate of hypoglycaemia in episodes per patient-years of exposure; Sita, sitagliptin; SU, sulphonylurea; %, proportion of patients with events.

The rate of overall observed confirmed hypoglycaemia per patient-year of exposure (PYE) was 3.07 episodes for IDeg and 1.26 episodes for Sita; the estimated rate ratio (ERR) (IDeg/Sita) was 3.81 (2.40; 6.05)_95% CI_ (Table 3). The proportions of subjects with confirmed hypoglycaemic episodes were higher with IDeg [42.5% (96/226) of subjects] compared with Sita [12.7% (29/228) of subjects]. For subjects that were not treated with SU/Pio, the rates for confirmed hypoglycaemic episodes were 1.92 episodes per PYE for IDeg and 0.0 for Sita (Table 4).

The rate of nocturnal confirmed hypoglycaemic episodes per PYE was 0.52 for IDeg and 0.30 for Sita; the ERR (IDeg/Sita) of 1.93 (0.90; 4.10)_95% CI_ (Table 3). In the IDeg group, 12.8% (29/226) of subjects reported nocturnal confirmed hypoglycaemic episodes compared to 5.7% (13/228) of subjects in the Sita group.

#### Adverse events

Overall rates of AEs and serious adverse events (SAEs) were similar for IDeg and Sita with no specific patterns or clustering. Similar proportions of subjects reported AEs [62.4% (141/226) of subjects] in the IDeg group and [63.2% (144/228) of subjects] in the Sita group. Slightly more subjects in the IDeg group reported AEs leading to withdrawal [3.9% (9/229) of subjects in the IDeg group and 0.9% (2/229) of subjects in the Sita group]. The most frequently reported AEs in both treatment groups were headache, diarrhoea, nasopharyngitis and nausea. The majority of AEs were mild or moderate and only a few of the AEs in either treatment group were severe. The rate of severe AEs and the rate of AEs possibly or probably related to trial product (IDeg/Sita) were similar between the two treatment groups. The majority of subjects in both treatment groups recovered from the AEs. Few subjects reported injection-site reactions with IDeg [4.4% (10/226) of participants], and the rate was 15 events per 100 PYE. All of the reported injection-site reactions were mild; none were reported as SAEs or led to discontinuation of the trial product.

A total of 17 SAEs were reported by 14 subjects (6.2%) in the IDeg group while 10 SAEs were reported by 10 subjects (4.4%) in the Sita group. The rates of SAEs were 17 and 10 events per 100 PYE with IDeg and Sita, respectively. No SAE was reported at a frequency ≥1% in either treatment group. One subject had a fatal SAE [myocardial infarction (day 82, age 58/male)] in the IDeg group, and investigators determined that all SAEs were unlikely to be related to trial products. No SAEs were considered by the investigator to be related to the injection devices in the IDeg group. The rate of cardiovascular events suspected to be related to acute coronary syndrome, stroke or cardiovascular death (major cardiovascular events) was similar between treatment groups: 0.03 (IDeg) and 0.03 (Sita) events per PYE.

#### Body weight

The observed change in mean body weight was higher with IDeg (+2.28 kg) than with Sita (−0.35 kg), with an ETD (IDeg−Sita) of 2.75 kg (1.97; 3.54)_95% CI_. The mean (s.d.) body weight at baseline and at end of trial was 83.9 kg (19.3) and 86.2 kg (20.0) in the IDeg group and 86.1 kg (19.8) and 85.8 kg (20.1) in the Sita group, respectively.

#### Other safety-related issues

No differences were noted in laboratory measurements, physical examination, ECGs and fundoscopy. After 26 weeks of treatment, there was no change in the mean blood pressure in the IDeg group (baseline; 129/78 mmHg and week 26; 129/78 mmHg), while there was a decrease in mean systolic blood pressure in the Sita group (baseline; 130/80 mmHg and week 26; 127/79 mmHg).

## Discussion

In most patients, the progressive deterioration of glucose control in type 2 diabetes increases the need for multiple additive therapies in order to attain recommended glycaemic targets. For patients where initiation of insulin would result in both short- and long-term added benefits with regard to glycaemic control, efforts need to be made to illustrate the direct benefits in efficacy that insulin has over currently available OADs while also trying to break down the known barriers to initiate insulin 18–20. The results from the present trial demonstrated that IDeg dosed at any time of day, with the possibility of adapting the injection time from day to day, was well tolerated and effectively improved glycaemic control in individuals with type 2 diabetes inadequately treated with 1–2 OADs. The population studied consisted of middle-aged, overweight or obese individuals with diabetes duration of∼8 years. The majority of individuals were treated with two OADs (metformin and SUs) at study entry, thus the population mimics a prevalent group of patients seen in everyday clinical practice. Treatment with IDeg lowered HbA1c by 0.43%-points more than did Sita, and the odds of achieving the goals of HbA1c < 7% (<53 mmol/mol) and HbA1c ≤ 6.5% (≤48 mmol/mol) were 60 and 98% higher with IDeg than with Sita, respectively. The 9-point SMPG profile showed that IDeg was also more effective than Sita in reducing both fasting and postprandial PG. In accordance with the ADA/EASD position statement, none of the mean level of postprandial PG in the IDeg group exceeded 180 mg/dl 9. The efficacy results seen in this study are in general agreement with previous results seen for both IDeg and Sita. In insulin-naïve subjects treated with 1–2 OADs, IDeg has been shown to effectively reduce HbA1c with non-inferiority to IGlar 21–23. When added to metformin, Sita has been shown in earlier studies to reduce HbA1c between 0.6 and 1.0% from baseline levels of 7.5–8.7% (58–72 mmol/mol) over 6–12 months’ therapy 24.

One of the greatest concerns for individuals with diabetes is the risk of hypoglycaemic episodes and, in particular, those occurring during sleep (nocturnal hypoglycaemia). With superior glycaemic control of IDeg compared with Sita, it was expected that the rate of hypoglycaemic episodes would be higher, as turned out to be the case. As the rate of hypoglycaemia in general was low in both treatment groups in this trial, in particular during the night, the rate of nocturnal confirmed hypoglycaemia was not shown to be statistically different. Moreover, no clustering of hypoglycaemic episodes at any time-point during the trial was observed. It is well known that SU use can be associated with higher risk of hypoglycaemia. As three quarters of all subjects entering the study were continuously treated with an SU/glinide in addition to trial drugs, a *post hoc* analysis was performed in order to analyse if the use of concomitant OADs affected the incidence of confirmed hypoglycaemia. As expected, in both treatment groups the *post hoc* analysis identified a marked decline in the number of hypoglycaemic episodes in individuals not treated with SUs/glinides.

In accordance with previous studies 24, no weight gain was observed with Sita, whereas a small weight gain was observed with IDeg, in similar magnitude as is commonly found with initiation of basal insulin treatment 25. Flexibility in dosing times may be of importance for patients. Data from a large survey have shown that approximately one third of patients report insulin omission/non-adherence on average 3 days within the last month due to being, for example, too busy, travelling or stressed 18. In this study, 42% of subjects treated with IDeg chose to change the injection time of their basal insulin at least once. This depicts the advantage of the option of flexibility when needed in a clinical context.

Limitations to the trial include the open-label design but, as the trial drugs were administered as injection (IDeg) and as oral agent (Sita), a blinded study was not an option. Poor control at baseline [mean baseline HbA1c: 8.8–9.0% (73–75 mmol/mol) and mean baseline FPG: 9.4–9.9 mmol/l (169–178 mg/dl)] may favour the addition of IDeg, which specifically targets FPG, compared with Sita, which has more of an effect on postprandial glucose.

In conclusion, the results from this trial demonstrated that the basal insulin, IDeg, as add-on to 1–2 OADs dosed once daily at any time of the day, was superior to Sita in terms of improving glycaemic control as measured by reduction in HbA1c in subjects with type 2 diabetes. Although hypoglycaemia rates are naturally higher when treating with insulin, the marked improvements in efficacy and, in the case of IDeg, the possibility of flexibility with regard to dose timing when needed, support the benefits of earlier initiation of basal insulin in relevant patient groups.
